# Phase Separation in the Nucleus and at the Nuclear Periphery during Post-Mitotic Nuclear Envelope Reformation

**DOI:** 10.3390/cells11111749

**Published:** 2022-05-25

**Authors:** Klizia Maccaroni, Mattia La Torre, Romina Burla, Isabella Saggio

**Affiliations:** 1 Department of Biology and Biotechnology, Sapienza University, 00185 Rome, Italy; klizia.maccaroni@uniroma1.it (K.M.); mattia.latorre@uniroma1.it (M.L.T.); romina.burla@uniroma1.it (R.B.); 2 CNR Institute of Molecular Biology and Pathology, 00185 Rome, Italy; 3 Institute of Structural Biology, Nanyang Technological University, Singapore 639798, Singapore; 4 School of Biological Sciences, Nanyang Technological University, Singapore 639798, Singapore

**Keywords:** liquid–liquid phase separation, post-mitotic nuclear envelope, telomeres, chromatin organization, nuclear condensate

## Abstract

Membrane-enclosed organelle compartmentalization is not the only way by which cell processes are spatially organized. Phase separation is emerging as a new driver in the organization of membrane-less compartments and biological processes. Liquid–liquid phase separation has been indicated as a new way to control the kinetics of molecular reactions and is based on weak multivalent interactions affecting the stoichiometry of the molecules involved. In the nucleus, liquid–liquid phase separation may represent an ancestral means of controlling genomic activity by forming discrete chromatin regions, regulating transcriptional activity, contributing to the assembly of DNA damage response foci, and controlling the organization of chromosomes. Liquid–liquid phase separation also contributes to chromatin function through its role in the reorganization of the nuclear periphery in the post-mitotic phase. Herein, we describe the basic principles regulating liquid–liquid phase separation, analyze examples of phase separation occurring in the nucleus, and dedicate attention to the implication of liquid–liquid phase separation in the reorganization of the nuclear periphery by the endosomal sorting complexes required for transport (ESCRT) machinery. Although some caution is warranted, current scientific knowledge allows for the hypothesis that many factors and processes in the cell are yet to be discovered which are functionally associated with phase separation.

## 1. Liquid–Liquid Phase Separation in the Cell

**Figure 1:**
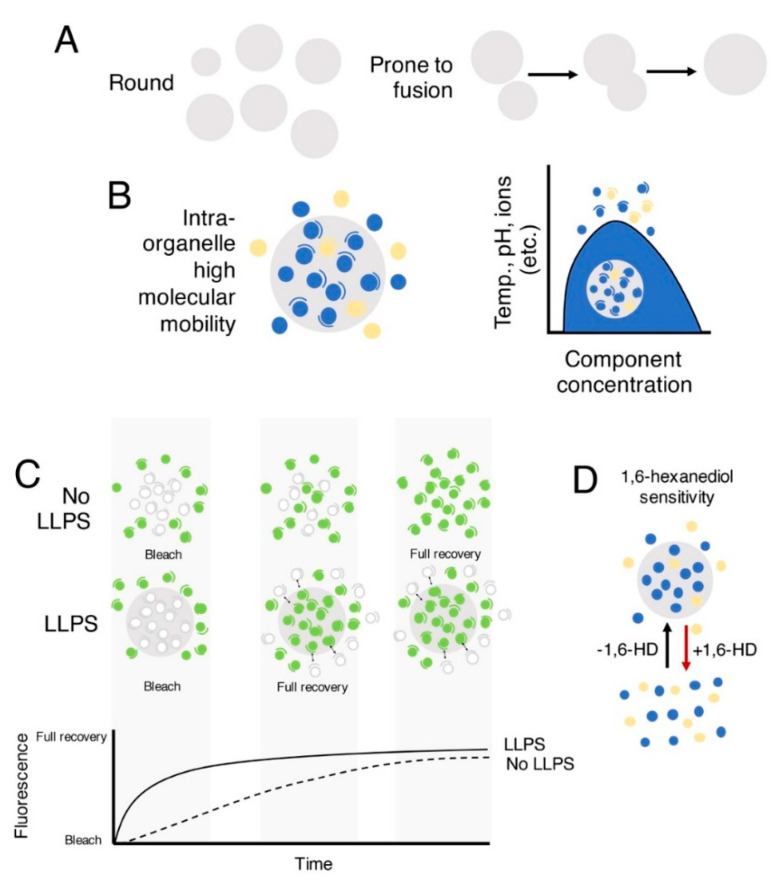
Features shared by LLPS condensates. (**A**) LLPS condensates (gray circles) must be round and prone to coalesce. (**B**) LLPS condensates depend on the concentration of the molecules (blue and yellow circles, different types of molecules) composing them and on environmental factors that influence their formation and dissolution. (**C**) One system used to study LLPS is FRAP, a technique quantifying fluorescence recovery kinetics after bleaching. The figure schematized the comparison of the recovery time for full photobleaching of an LLPS-dependent and an LLPS-independent organelle. In LLPS, fluorescence recovery has been often suggested to proceed faster. (**D**) LLPS condensates can be sensitive to the 1,6-hexanediol, which disrupts weak hydrophobic interactions.

Phase separation is a phenomenon based on the concept that a mixture of molecules can spontaneously separate into two phases that differ in their composition and in the local concentration of specific factors [1]. Liquid–liquid phase separation (LLPS) is promoted by weak multivalent interactions and it occurs in the cell in a way that resembles oil and water de-mixing [2]. However, recent studies have demonstrated that this familiar and widely used example of phase separation is an over-simplification with respect to LLPS occurring in cellular systems, in which the viscoelastic nature of the intracellular environment exerts an important control on the growth dynamics and on size of LLPS condensates [3,4]. Phase separation can generate compartmentalized biomolecular condensates and is now considered to be one of the elements that contribute to control of intracellular processes [5,6,7,8]. The advantage for the cell in having some of its processes regulated by phase separation lies in the possibility to economically and dynamically sub-compartmentalize molecules [2]. Major efforts have been dedicated to the definition of the properties required for an organelle to imply LLPS as the causative principle in their formation and functional regulation. Although additional understanding is still needed, it is possible to identify some features that are frequently shared by LLPS-based organelles. Firstly, the organelle must be round and prone to fusion (two properties that depend on surface tension), and characterized by intra-organelle high molecular mobility (Figure 1A). Secondly, the properties of the organelle must depend on the concentration of its components and on some environmental conditions such as ions, temperature, and pH. These influence LLPS-based organelle formation and dissolution by changing the critical local concentration at which the molecules phase-separate (Figure 1B). Thirdly, the assembly and disassembly of organelles based on LLPS should occur under physiological conditions, and their dynamics are usually studied both in vitro and in vivo. However, some caution is needed when interpreting the in vitro results about LLPS organelle formation. Indeed, the phase separation properties seen in vivo are often different from those observed in vitro, and the in vitro formation of some LLPS condensate observed in cells is based on specific conditions, specific protein modifications, or specific partners [9,10,11,12]. One of the most-used techniques to define and study LLPS organelles is fluorescence recovery after photobleaching (FRAP). In full-FRAP, a fast recovery kinetics of the entire bleached organelle from the non-bleached surrounding area has been considered indicative of LLPS (Figure 1C). It should be added, however, that it was recently outlined that a fast FRAP can also have different interpretations. This is especially significant when it is applied to small or fast-moving endogenous intracellular structures. Full-FRAP could be integrated by half-FRAP if the organelle is big enough to be partially bleached. In half-FRAP, the recovery rate could give information about the internal mobility of the molecules of the organelle [13,14]. Lastly, some LLPS membrane-less organelles have been found to be sensitive to the chemical compound 1,6-hexanediol [15,16] (Figure 1D). It should also be underlined that recent studies have demonstrated that not all LLPS-based condensates are sensitive to 1,6-hexanediol and that its use in vivo may change membrane permeability and may thus be associated with artifacts [16].

In the cell, a prototypic example of LLPS-induced intracellular membrane-less organelles are P granules in *C. Elegans*. These are a class of germline-specific RNA containing perinuclear granules. P granules function in development of gametes where, in association with the nuclear pore complex, they control the presence of non-germline transcripts [17]. The first descriptions of phase separation date back to 1800 (reviewed in [18]). The nucleolus was one of the first membrane-less organelles described for having liquid-like properties [19] and was later proposed to be a coacervate of histone-like proteins, positively charged, and nucleotide, negatively charged [20]. The work of Brangwynne and collaborators on LLPS nature of P granules [21] successively promoted the development of the field and the discovery of the properties of phase-separated condensates. Indeed, it was shown that the vesicles composing P granules can fuse with adjacent vesicles and switch between a spherical-condensed and a soluble form [21]. FRAP microscopy experiments, in which half of P granules expressing a fluorescently tagged constituent protein (i.e., GFP:PGL-1) was bleached, showed a rapid recovery time and a simultaneous decrease in the fluorescence of the adjacent unbleached regions, suggesting a high internal mixing diffusion [21]. Other LLPS membrane-less organelles in the cell are Cajal bodies [22], stress granules [23], nucleoli [24], and promyelocytic leukemia (PML) bodies [25]. These organelles, despite being involved in different fundamental cellular processes, have in common the property of concentrating together different elements required for their specific function. In particular, the Cajal bodies contribute to the modification and assembly of snRNPs [26], stress granules concentrate untranslated mRNPs that form from mRNAs stalled in translation initiation [27], and nucleoli are the basic central structures for the synthesis of ribosomal RNA molecules [28]. Finally, PML bodies are nuclear-matrix-associated aggregates that contain many different factors and operate in multiple processes. A pivotal functional element in PML activity is exerted via a specific post-translational modification, i.e., sumoylation [29]. LLPS has also been suggested to be a primordial DNA condensation mechanism such as that described in bacteria nucleoids, which behave as fluids [30]. In eukaryotes, this is observed in mitochondrial DNA [31].

## 2. Factors Contributing to Liquid–Liquid Phase Separation

**Figure 2:**
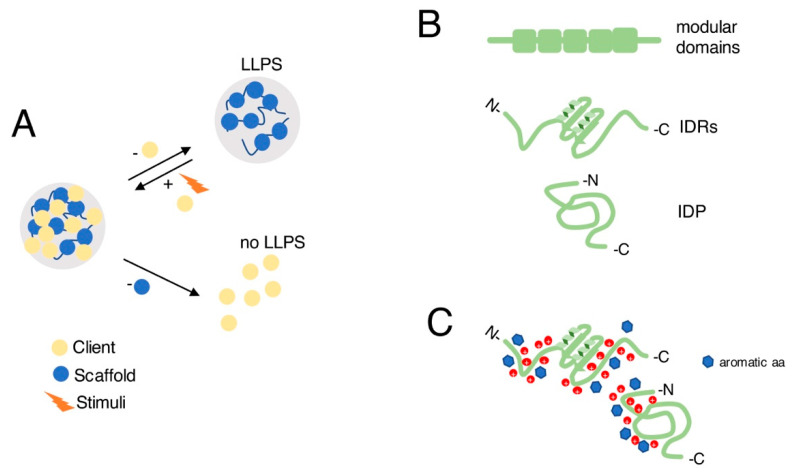
Factors contributing to LLPS condensates formation. (**A**) Scaffold (blue circles) and client (yellow circles) components of LLPS condensates and their role in condensate formation. Clients bind scaffold elements in a regulated way, and their composition varies in response to stimuli (orange lightning). Clients are dispensable for the assembly of the condensate; otherwise, scaffold elements are necessary to assemble a LLPS condensate. (**B**) Three classes of proteins contribute to LLPS: proteins containing repetitions of modular domains (green boxes); proteins that contain ordered and intrinsically disordered regions (IDRs; green); intrinsically disordered proteins (IDPs). (**C**) Examples of non-covalent pi-cation interactions between aromatic residues (blue hexagons) and cations (red circles) of positively charged amino acids that could account for LLPS formation by proteins with IDRs.

The factors that contribute to generation of cell condensates share common chemical and physical characteristics, including amino acid compositions, enrichment in disordered regions, multivalency, and presence of highly charged regions. These are responsible for the behavior of LLPS and are well-explained by the concept of scaffold and client molecules [32]. The scaffold (protein or nucleic acid), as the name suggests, is responsible for the structural integrity of the condensates, while clients, despite often being the most abundant elements of the condensate, are dispensable for the assembly and bind to molecules of the scaffold in a regulated way (Figure 2A). The clients can diffuse and change rapidly. The type of clients in the condensate varies in response to specific stimuli and defines the function and role of the condensates, enabling a rapid response [22]. In PML bodies, the PML protein acts as a scaffold that is necessary for the recruitment of other proteins such as nuclear body protein Sp100 [33,34], which acts as a client. The post-translational modification of scaffold proteins, for example by phosphorylation, can regulate the properties of LLPS condensates. In PML bodies, the phosphorylation of its component SUMO1 enhances the interaction with the protein SIM. Consistently, mutations in the phosphoserine SIM residues decrease the interaction of SIM with SUMO1 in PML bodies [35]. Another example is the non-covalent interaction of the Daxx protein with SUMO in PML bodies, which is regulated by phosphorylation [36].

In general terms, the proteins that can contribute to LLPS can be subdivided into three classes of proteins (Figure 2B). The first class is characterized by the presence of a sequence including repetitions of the same domain. Such modular domains render the formation of condensates thermodynamically favorable and stabilize the weak non-covalent interactions within condensates, supporting the formation of larger complexes. Indeed, the oligomerization in larger complexes enhances the weak, non-covalent interaction between molecules, reducing their solubility and favoring phase separation [37]. The multivalent proteins Nephrin, Nck, and N-WASP, for example, which are part of an actin-regulatory signaling pathway, associate into a larger complex thanks to the interaction between residues phosphorylated tyrosine residues of Nephrin and the SH2 domain of Nck and between SH3 domain of Nck and proline-rich motifs (PRMs) of N-WASP. This association is sufficient to induce phase separation in vitro [38]. The second main class of macromolecules that is commonly involved in LLPS is that of intrinsically disordered proteins (IDPs), which includes proteins composed of amino acids that are not expected to organize into a specific 3D structure. An example of IDPs is that of the structural microtubule-associated proteins (MAPs). Type-1 structural MAPs are predicted to be highly disordered and they exploit a highly basic N-terminal domain to associate with the negatively charged surface of microtubules [39]. Another class of proteins includes polypeptides that contain ordered and intrinsically disordered regions (IDRs), which often have low sequence complexity and enriched in polar amino acids such as serine, tyrosine, glutamine, and asparagine [40]. This low sequence complexity is responsible for LLPS and offers a scaffold by which many non-covalent interactions are possible especially between nucleic acids and proteins. The non-covalent interactions mediated by pi-electrons in non-aromatic residues, pi-pi between two aromatic residues, and pi-cation interactions between aromatic residues and cations of positively charged amino acids, account for phase separation of proteins with IDRs (Figure 2C) [41]. An example of this principle is offered by the DEAD-box helicase in P granules. This protein is characterized by an IDR composed of positively charged amino acids at both its C and N termini that account for its ability to self-associate inter- and intra-molecularly via weak electrostatic interactions. Moreover, the IDR N terminus can undergo post-translational modifications that weaken the stability of the condensate and putatively regulate these particles [42,43].

## 3. Liquid–Liquid Phase Separation in the Nucleus

**Figure 3:**
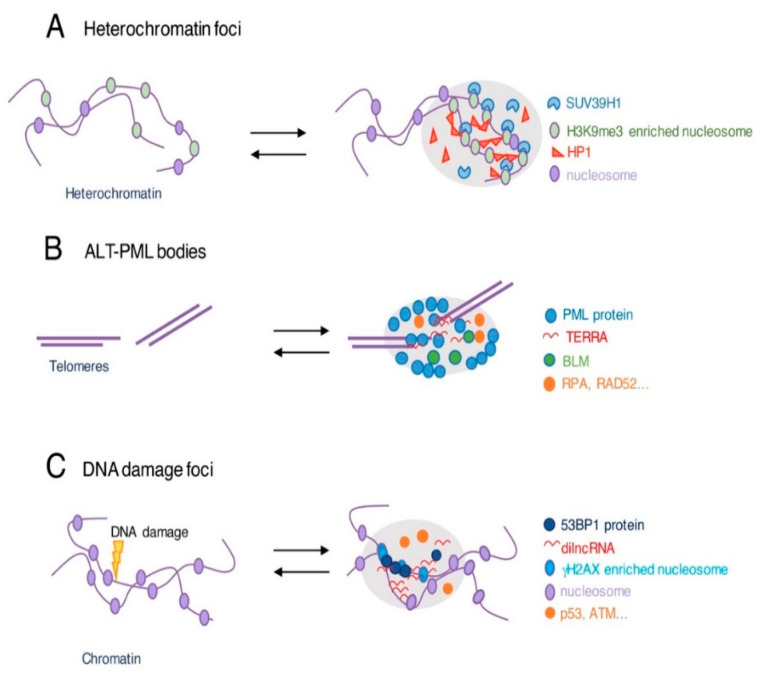
Examples of LLPS condensates in the nucleus. (**A**) LLPS based heterochromatin organization acts at chromatin regions enriched in H3K9me3 histone modifications (green) bound by proteins containing chromodomains, such as HP1 (red) and SUVAR39H1 (light blue), increasing the compaction of the chromatin. (**B**) ALT-associated PML bodies (APBs) are formed by LLPS clustering telomeres (purple), PML protein (blue), and other factors such as BLM (green), DNA damage response proteins (orange), and the long non-coding RNA associated with telomeres, (TERRA; red). (**C**) At site of DNA damage, foci are promoted by 53BP1 (blue) and by the long non-coding RNAs (dilncRNA; red), which increases the concentration of DNA damage response proteins (orange) such as p53.

In the nucleus, chromatin can be viewed as a platform on which liquid droplets assemble via LLPS. If LLPS is considered the main mechanism of phase separation in the nucleus, chromatin may also undergo a second type of phase separation, named polymer-polymer phase separation (PPPS). In PPPS, links occur between different regions of chromatin fibers, thereby inducing a collapse of chromatin into a globular phase [44]. In LLPS, on the other hand, multivalent interactions occur among soluble molecules that bind to chromatin. In this case, nuclear bodies assemble on chromatin. LLPS and PPPS have been proposed as two different, but not mutually exclusive, mechanisms to explain the formation of phase-separated chromatin compartments [44,45]. We will focus here on significant examples of LLPS at chromatin, but for an in-depth description of the implications of LLPS in the overall regulation of gene expression, we address the reader to other excellent publications [46,47]. A well-documented example of LLPS-based organization of chromatin is that occurring in its heterochromatic portions [48,49] (Figure 3A). LLPS acts here via histones that bind proteins which possess chromodomains, such as SUV39H1 or HP1. These chromodomain proteins are conserved in yeast, *D. melanogaster*, and humans [50,51,52]. Functionally, this process contributes to improving the compactness of heterochromatin. LLPS is also implicated in the functional organization of centromeres. The heterochromatin of the inner centromere must deal with the complexity of the multiple functions of these chromosomal structures, including the maintenance of cohesion of sister chromatids and the resistance to microtubule pulling forces during cell division. LLPS has been suggested as a biophysical strategy to compartmentalize these different events. This is achieved via sub-regions of the chromosome passenger complex (CPC) and its multiple components including INCENP, survivin, and borealin. Indeed, CPC factors have been shown to possess several characteristics associated with LLPS, both in vitro and in vivo, [53] such as the capability to form condensates at low salt and at high protein concentrations. LLPS of the CPC condensates is further conditioned by centromeric histone variants and by HP1α, because their phosphorylation is proposed as the initial nucleation driving CPC phase separation to the inner centromere [54,55,56,57].

Intranuclear LLPS affecting chromatin organization is also observed at telomeres (Figure 3B). Specifically, LLPS at telomeres has emerged in association with the alternative lengthening of telomers (ALT), a telomerase-independent, recombination-dependent mechanism of telomere lengthening shown by telomerase-negative tumors [58]. Telomeres undergoing ALT are clustered to form the so-called ALT-associated PML bodies (APBs) [59,60]. In these membrane-less organelles, telomeric and non-telomeric proteins are recruited to the same site to generate foci that, over time, become larger, rounder, and brighter and acquire the characteristics associated with liquid condensates [61]. LLPS clustering of telomeres in APBs allows the concentration of factors responsible for telomeric DNA synthesis and elongation. The high concentration of BLM helicase, for example, accounts for the generation of ssDNA that is responsible for ALT induction and telomere replication [62]. APB bodies also concentrates DNA damage response factors [61] and long non-coding RNA TERRA, which can autonomously organize into distinct foci [63], in a condensate organized around telomeric chromatin [64]. The implication of TERRA in LLPS leads to the speculation that the organization and function of telomeres may imply phase separation, even outside of the framework of APB bodies and ALT positive cells. This could be substantiated by the particular structural nature of telomeres. Indeed, they are composed of DNA repetition elements (TTAGG repeats), they are organized into a putative G-quadruplex structure which is prone to phase separation [65,66], and the complex of the shelterin factors protecting telomere ends has a (complementary) repetitive nature [67]. Consistent with this hypothesis, a recent work describes telomere behavior as liquid-like condensates, at least in vitro [68]. This work shows that telomeric DNA acts as a scaffold to favor the shelterin-mediated phase separation. In this process, a predominant role is played by TRF1 and TRF2, two main components of the shelterin complex. These factors show a propensity to phase separate in vitro that depends on both their IDR and their dimerization domains [68]. The demonstration that this process also happens in vivo is yet not obtained.

A further case of LLPS in the interphase nucleus is the pathway that allows signaling of DNA breaks which depends on the recruitment of ATM and 53BP1 at damaged DNA sites (Figure 3C). Protein recruitment induces the formation of subnuclear compartments that protect DNA from further enzymatic degradation [69,70,71]. The factor 53BP1 can phase-separate, both in vitro and in vivo. Consistently, osmotic stress and elevated salt concentration impair the formation of 53BP1 subnuclear compartments on DNA. Moreover, time-lapse microscopy has shown that 53BP1 can form droplets and is sensitive to 1,6-hexanediol [72]. In this process, damage-induced long non-coding RNAs synthesized at double-strand breaks in DNA promote the concentration of DNA damage response proteins into foci, thus contributing to the LLPS process [73].

New tools are being currently developed to further define the properties of phase separation that will help to precisely assess whether the indicated or suggested events of phase separation occur in the cell nucleus, and, importantly, how they precisely contribute to the regulation of chromatin function.

## 4. Liquid–Liquid Phase Separation in the Post-Mitotic Reforming Nucleus

**Figure 4:**
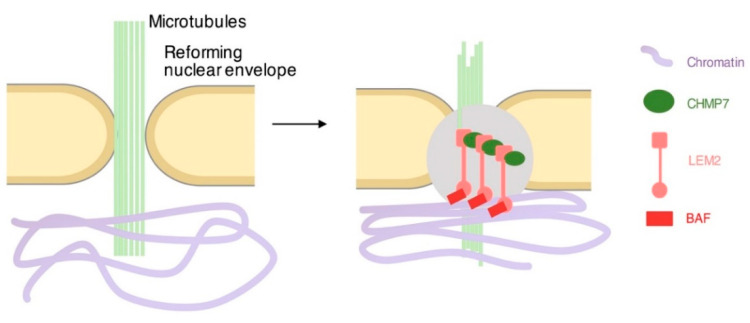
LLPS at the reforming nuclear envelope. During nuclear envelope reformation, membranes are reassembled and nuclear holes surrounding remaining microtubules are sealed by ESCRT machinery. The recruitment of CHMP7 (green) and ESCRT III nucleation is promoted by LLPS of LEM2 (pink) that interacts with BAF (red), which in turn interacts with chromatin (purple) and with CHMP7 through its winged-helix domain.

During cell division, higher eukaryotic cells pass through nuclear envelope breakage and nuclear envelope reforming. The breakage permits the association of chromosomes with the spindle microtubules, which in turn contributes to correct segregation of genetic material into daughter cells [74]. The reformation is crucial to recreate the separation of nuclear chromatin from the cytoplasm and contributes to its reorganization. In the post-mitotic nuclear envelope reforming phase, membrane fragments are reassembled and nuclear holes in the membrane are sealed [75,76,77]. This happens in parallel and in conjunction with the compartmentalization of chromatin territories in the nucleus. Interestingly, in human cells, in the early post-mitotic stage, telomeres are tethered at the nuclear envelope which is reforming. This acts as an early driver for post-mitotic chromatin reorganization, including the positioning of heterochromatin at the nuclear periphery and the reorganization of lamina-associated domains (LADs) [78]. A recent study has suggested that nascent transcripts also play a role in chromatin organization, thereby contributing to the formation of a dynamic ribonucleoprotein scaffold that promotes the formation of an accessible chromatin environment, which is reflected in the cytological decondensation of chromatin [79]. During this delicate phase of nuclear compartmentalization, a specialized machinery is required: the endosomal sorting complex required for transport (ESCRT) [80]. This machinery is composed of multiple protein macro-complexes and was initially characterized for its activity in endosome trafficking. In this process, it sorts ubiquitylated proteins and contributes to the formation of intracellular multivesicular bodies, which are destined for lysosomes [81]. The ESCRT machinery has been also mechanistically implicated in cell division. In particular, the machinery is required for the abscission of the bridge linking the two daughter cells in the final stages of cell division [82,83]. The analysis of this process offers paradigmatic images of the stepwise formation of the macro-complex of ESCRT machinery [84,85]. This starts with the concentration of factors belonging to the ESCRT I complex at the center of the bridge. ESCRT II and III subunits of the machinery, or, in an alternative route, protein ALIX, are then recruited. The positioning of the multimeric rings of ESCRT III subunits finalizes the assembly of the machinery [86] and is essential in the very final stages, which depend on the enzyme spastin and the ATPase VPS4 [87,88]. In the reforming nuclear envelope, the ESCRT machinery involves the activity of the ESCRT III subunit CHMP2A and ESCRT III CHMP4B. In addition to these ESCRT III subunits, the ESCRT II-III hybrid factor CHMP7, together with the p97 complex member ubiquitin fusion and degradation 1 (UFD1), provide physical and mechanistic support [89]. LLPS has been suggested to be involved in the functional assembly of these factors. The process is bridged with chromatin via a factor named LEM2 and its interactor BAF [89,90]. In particular, the ESCRT transmembrane adaptor LEM2 enriches together with the ESCRT CHMP7 at the chromatin disk periphery [76]. Here, it contributes to create a physical platform around residual microtubule fibers that is functional to the recruitment of the other ESCRT subunits CHMP2A and IST1. These are needed, in turn, for the recruitment of spastin for microtubule severing and finalization of the entire process (Figure 4). The polymerization of the ESCRT machinery is favored by the ability of LEM2 to undergo LLPS [90]. Indeed, LEM2 contains a low-complexity domain (LCD) that can phase-separate. Experiments on this domain show that, in vitro, it spontaneously forms droplets that undergo fusion in physiological salt conditions [90]. Using super-resolution imaging, it is possible to observe LEM2 binding to microtubules. In vitro, photobleaching experiments show that LEM2 recovers the fluorescence in a co-axial manner, confirming a liquid-like behavior when in association with microtubules [90]. The LEM motif of LEM2 binds BAF [91,92,93], while the winged-helix domain of LEM2 activates the ESCRT-II/ESCRT-III hybrid protein CHMP7 to form ring shaped macro-complexes. At the end of anaphase, BAF, thanks to its dimerization activity and its high affinity for chromatin, mediates DNA-cross bridging that contributes to the formation of a compact and stiff chromatin surface that impedes the access of reforming membranes to the interior of bulk chromatin [94]. In addition to completing reformation post-mitotically, the ESCRT subunit CHMP7 contributes to repair of nuclear envelope ruptures that occur during interphase [95,96].

Recently, a new factor, named AKTIP, was discovered that intercepts the concepts of nuclear envelope, ESCRTs, and telomere function [97,98,99]. AKTIP deficiency generates telomere fragility and has the singularity as a telomeric protein to be enriched at the nuclear envelope [97,99,100]. AKTIP has sequence similarity with the protein TSG101, a tumor susceptibility gene that functions as ESCRT I in viral budding and cytokinesis [83], and AKTIP acts in association with ESCRTs in cytokinesis [98]. In vivo, AKTIP assembles into discrete foci at the nuclear envelope [97,98,99] and contains two disordered regions that may be potentially involved in LLPS. Given the above, it would be tempting to speculate that phase separation can contribute to AKTIP activities at the nuclear envelope. Extending this concept, it can be further speculated that LLPS-controlled events at the nuclear periphery have an impact on the organization and function of telomeres in the early post-mitotic stage [97,98,99].

## 5. Conclusions

The separation of functions is a crucial mechanism to correctly execute molecular processes within the cell. LLPS has been indicated as a means of acting on the kinetics of molecular reactions by influencing the stoichiometry of the molecules involved. In fact, the spontaneous formation of membrane-less organelles in which factors are condensed allows for faster activation of the specific mechanisms, as well as a faster way of dismantling these when needed. LLPS may also represent an ancestral means of controlling cell and genomic activities [101]. It has an important role in organizing discrete chromatin regions by extending the plasticity of chromatin function in higher eukaryotes [48] and is believed to play a role in DNA damage response. LLPS can have an impact on chromatin through its role in the reshaping of the nuclear envelope in the post-mitotic stage when chromatin territories are reorganized [90]. This is also the stage during which mammalian telomeres are transiently tethered at the nuclear envelope as a putative anchor point to reorganize chromatin [78]. Although additional understanding is needed to confirm the cell functions associated with phase separation, this process may redefine the relative role of multivalent weak interactions in cell biology.
